# Sanitation for Scotland

**Published:** 1897-05-29

**Authors:** 


### The Hospital

A Journal of

The Medical Sciences and Hospital Administration.

Vol. XXII.—No. 557.] [May 29, 1897.

### SANITATION FOR SCOTLAND.

It seems not unlikely that the progress of sanitary legislation is about to provide a sufficiently vexatious grievance for our fellow subjects on the north of the Border. A Public Health (Scotland) Bill is at present under the consideration of a Committee of the House of Commons, and a protest against certain of its provisions has been put forth, on behalf of the Society of Medical Officers of Health for that division of the kingdom, by eleven of the most eminent and most representative members of their body, including Dr. McVail, Sir Henry Littlejohn, and Dr. Russell. The ground of the protest is an alteration which has been introduced in Committee into the 22nd clause of the Bill, and which has been accepted by the Lord Advocate, to the effect that a local authority shall be empowered to take legal proceedings for the removal or rectification of the majority of nuisances, not only, as in the original draft, on the certificate of the Medical Officer, but also, and alternatively, on that of a "sanitary inspector." The effect of the alteration will be that as regards nine out of eleven classes of nuisances, every local authority will be able to proceed for their suppression on the certificate of a sanitary inspector as to what is injurious or dangerous to health; and this although the law requires no qualification of any kind for any person appointed as a sanitary inspector to any local authority, whether burghal or rural, in Scotland. It is, therefore, proposed to place every such unqualified person on the same footing with an officer who possesses not only a medical qualification, but a degree or diploma in public health. The two excepted classes of nuisances include works, manufactories, collections of rags and bones, and schoolhouses; with the absurd result that it will be necessary to obtain a certificate from a medical officer that a collection of rags or of bones is injurious to health, while a certificate from a sanitary inspector will suffice as to dwelling-houses, or as to wells and water supplies, although in many instances the latter, as a matter of course, cannot be safely pronounced upon without a considerable knowledge of bacteriology, of chemistry, and of geology.

We cannot wonder that the Scottish medical officers of health have lost no time in expressing their opinion of the consequences which would be likely to flow from thus placing ignorance upon the same level with knowledge; and they rightly lay stress upon the manner in which, if the clause as altered in Committee should become law, the public will be liable to be harassed and injured by the wholesale initiation of legal proceedings regarding sanitary matters under the certificates of unqualified officers. We have had in England ample experience of the readiness of meddlesome persons, rendered rash by ignorance, to speak as if with authority upon sanitary questions; and it would be difficult to forecast whether Scottish inspectors, under the proposed legislation, would be more likely to wield their authority in an arbitrary manner, or entirely to neglect duties which they would, in most cases, be manifestly incompetent to discharge.

The state of public opinion in relation to sanitary questions is believed to be less advanced in Scotland than in England; and, in these circumstances, the operation of an Act of Parliament should be such as to exert a beneficial educational influence. It is said that, not so long ago, a Scottish minister complained to the heritors of his parish of the insanitary condition of his manse, and urged that certain improvements should be effected without delay. Finding them unwilling to spend money for any such purpose, he applied to Sir Henry Littlejohn, one of the signatories of the protest above-mentioned, and obtained from him, after careful inspection of the premises, a certificate that their condition was dangerous to the inmates. The certificate was duly considered by the heritors, and a deputation from that body proceeded to the manse with their report. They admitted that Sir Henry had declared the state of the dwelling to be perilous, and then proceeded to say, "But yet, minister, on the whole, we think we'll risk it." With such a state of public feeling, it is exceedingly desirable that any legal proceedings for the protection of health should at first be such as speedily to justify themselves by their consequences, and gradually to teach the inhabitants of the localities affected that the sanitary authorities were good friends to them and to their children. Where an enlightened public opinion has been developed by time and by experience an occasional unwise or misdirected prosecution will do no harm; but, where there is no adequate sense of the evils produced by nuisances, any interference with them is likely to be resented by their proprietors as superfluous, even if not as vexatious, and should be confined to carefully selected cases, in which early and manifest good results can be obtained. A medical officer of health will constantly find it necessary, in such localities, to exhaust the resources of reasoning and persuasion before he attempts to put in force the legal powers to which he may be able to resort in the last extremity; and it will even frequently be expedient for him to shut his eyes to many conditions which he would like to change. What is wanted is a combination of knowledge with prudence and tact; and a proposal to commit the power of instituting legal proceedings to a vestry, or to the Scottish equivalent for it, on the certificate of a possibly, and even probably, ignorant subordinate official, is one that Parliament should hesitate to confirm. Perhaps, after all, the really saving condition, in the Bill as it now stands, may be that the vestry, after receiving the inspector's certificate, will be able to act or not at its discretion.

